# Apicomplexan phosphodiesterases in cyclic nucleotide turnover: conservation, function, and therapeutic potential

**DOI:** 10.1128/mbio.03056-23

**Published:** 2023-12-22

**Authors:** William J. Moss, Lorenzo Brusini, Ronja Kuehnel, Mathieu Brochet, Kevin M. Brown

**Affiliations:** 1 Department of Microbiology and Immunology, University of Oklahoma Health Sciences Center, Oklahoma City, Oklahoma, USA; 2 Department of Microbiology and Molecular Medicine, Faculty of Medicine, University of Geneva, Geneva, Switzerland; Ohio State University, Columbus, Ohio, USA

**Keywords:** phosphodiesterase, PDE, cyclic nucleotide, cGMP, cAMP, parasite

## Abstract

Apicomplexa encompasses a large number of intracellular parasites infecting a wide range of animals. Cyclic nucleotide signaling is crucial for a variety of apicomplexan life stages and cellular processes. The cyclases and kinases that synthesize and respond to cyclic nucleotides (i.e., 3′,5′-cyclic guanosine monophosphate and 3′,5′-cyclic adenosine monophosphate) are highly conserved and essential throughout the parasite phylum. Growing evidence indicates that phosphodiesterases (PDEs) are also critical for regulating cyclic nucleotide signaling via cyclic nucleotide hydrolysis. Here, we discuss recent advances in apicomplexan PDE biology and opportunities for therapeutic interventions, with special emphasis on the major human apicomplexan parasite genera *Plasmodium*, *Toxoplasma*, *Cryptosporidium*, and *Babesia*. In particular, we show a highly flexible repertoire of apicomplexan PDEs associated with a wide range of cellular requirements across parasites and lifecycle stages. Despite this phylogenetic diversity, cellular requirements of apicomplexan PDEs for motility, host cell egress, or invasion are conserved. However, the molecular wiring of associated PDEs is extremely malleable suggesting that PDE diversity and redundancy are key for the optimization of cyclic nucleotide turnover to respond to the various environments encountered by each parasite and life stage. Understanding how apicomplexan PDEs are regulated and integrating multiple signaling systems into a unified response represent an untapped avenue for future exploration.

## INTRODUCTION

The phylum Apicomplexa encompasses a large number of single-celled intracellular parasites that threaten human and animal health worldwide. Of these, *Plasmodium* poses the greatest risk to human health, causing 247 million cases of malaria resulting in 619,000 deaths in 2021 (1). Even more prevalent but less deadly, *Toxoplasma gondii* infects a staggering one-third of humans and causes toxoplasmosis, which can be life-threatening in immunocompromised patients or during fetal development (2). *Cryptosporidium* is a waterborne parasite associated with outbreaks and is a leading cause of diarrheal death in infants and young children (3, 4). *Theileria*, *Babesia*, *Eimeria, Neospora*, and *Besnoitia* cause considerable economic loss due to the infection of livestock or poultry (5). *Babesia* is also notable for causing zoonotic illness in humans resembling a mild form of malaria (6). Apicomplexans continue to devastate their hosts despite immune recognition and therapeutic intervention due to robust environmental sensing, dynamic signaling, and adaptive lifecycles.

Apicomplexa show fascinating lifecycles with multiple differentiation steps into morphologically distinct forms in a broad range of hosts and environments. Multiple signaling pathways have been shown to be essential for these cellular and developmental changes that support apicomplexan parasitism (7). This includes the intracellular secondary messenger cyclic nucleotides: 3′,5′-cyclic adenosine monophosphate (cAMP) and 3′,5′-cyclic guanosine monophosphate (cGMP). Both messengers are found in organisms across the eukaryotic tree where they have been shown to play a myriad of functions. Despite the functional diversity of cAMP and cGMP secondary messengers across eukaryotes, cyclic nucleotide signaling universally relies on fine-tuning of local intracellular levels to activate effectors, including the cAMP-dependent protein kinase (PKA) or cGMP-dependent protein kinase (PKG). Cyclic AMP and cGMP are synthesized by adenylyl- and guanylyl cyclases from purines ATP and GTP, respectively (8). Since apicomplexan parasites are purine auxotrophs (9), the availability of purines in the host or extracellular environment could impact cyclic nucleotide signaling, but it has not been investigated in these organisms. When cellular cyclic nucleotide concentrations reach threshold levels, they stimulate their cellular effectors. The number of cellular effectors in apicomplexans is relatively limited and includes PKA, PKG, and a cAMP-binding protein called “exchange protein directly activated by cAMP” (10). To fine-tune cyclic nucleotide levels, cyclic nucleotides are degraded by 3′,5′-cyclic nucleotide phosphodiesterases (PDEs) (11). Recent works have highlighted the crucial role of apicomplexan PDEs in regulating a variety of critical cellular processes, including the development and dissemination of various lifecycle stages.

The 3′,5′-cyclic nucleotide PDE superfamily is divided into two major classes (12). Class I PDEs (pfam: PF00233) contain the H-D-[LIVMFY]-x-H-x-[AG]-x (2)-[NQ]-x-[LIVMFY] signature and are well conserved in eukaryotes, with a few examples also found in bacteria and archaea. Class II PDEs (pfam: PF02112) contain the H-x-H-L-D-H-[LIVM]-x-[GS]-[LIVMA]-[LIVM] (2)-x-S-[AP] signature and are mostly found in bacteria and fungi, with rare examples in amoeba and slime molds. All apicomplexan PDEs are Class I, but the number of PDEs varies widely. For example, piroplasms like *Babesia* and *Theileria* encode two PDEs. *Cryptosporidium* has three PDEs, while *Plasmodium* has four PDEs. On the other hand, coccidians like *Toxoplasma* and *Neospora* have 18 PDEs. Disparity in PDE numbers prompts the question, is the over-representation of PDEs in coccidia due to recent expansion or the retention of otherwise redundant genes in all other Apicomplexa?

**Fig 1:**
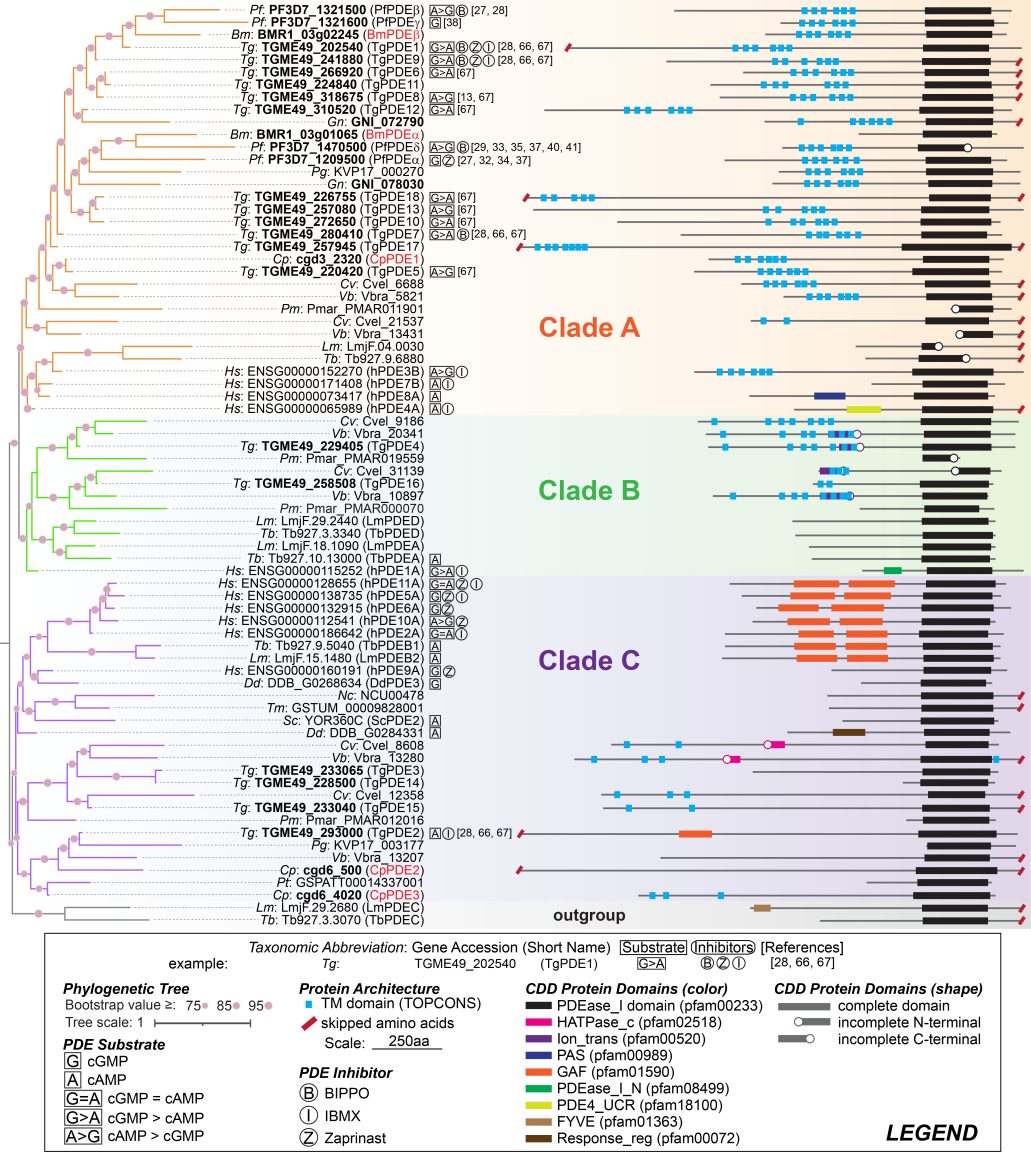
Apicomplexan PDEs evolved from three eukaryotic lineages. Pruned tree of a maximum likelihood tree inference, based on an alignment of eukaryotic PDEs. Support beside nodes represents rapid bootstrap (2,000 replicates) support. Full tree can be found in File S1. The eukaryotic PDEs are labeled by an italicized taxonomic abbreviation, then by the gene accession number (bold indicates apicomplexan PDEs), and then its short name in parenthesis (red font indicates purposed names from this review). Boxed letters represent the substrate preference of the PDE. Circled letters represent a PDE inhibitor that reduces PDE hydrolysis activity. Numbers in brackets correspond to references the data were derived from, if any. Human PDE substrate data were derived from references 14, 15, and human PDE inhibitor data from references (16–19). Other organism’s nomenclature, substrate, or enzyme data were derived from the respective organism database website. Whole protein sequences were put through TOPCONS (20) to identify transmembrane (TM) domains, or the CDD (21) to identify pfam protein domains. Colored lines represent different pfam protein domains, and a circle is placed on the side of the domain line if CDD determined the domain was incomplete. Red angled lines represent an unspecified amount of amino acids removed from the schematic for brevity. Protein architecture was subjectively aligned by the PDEase_I domain and the length and location of domains are to scale for each protein, performed on Adobe Illustrator. Taxonomic abbreviations: *Bm*, *Babesia microti*; *Cp*, *Cryptosporidium parvum*; *Cv*, *Chromera velia*; *Dd*, *Dictyostelium discoideum*; *Gn*, *Gregarina niphandrodes*; *Hs*, *Homo sapiens*; *Lm*, *Leishmania major*; *Nc*, *Neurospora crassa*; *Pf*, *Plasmodium falciparum*; *Pg*, *Porospora cf. gigantea B*; *Pm*, *Perkinsus marinus*; *Pt*, *Paramecium tetraurelia*; *Sc*, *Saccharomyces cerevisiae*; *Tb*, *Trypanosoma brucei*; *Tg*, *Toxoplasma gondii*; *Tm*, *Tuber melanosporum*; and *Vb*, *Vitrella brassicaformis*.

To explore the relationships between apicomplexan PDEs, within the broader context of eukaryotic PDEs, we extracted a set of 700 PDE sequences from a wide variety of eukaryotic organisms, representing each of five to six major eukaryotic supergroups. We analyzed sequences by phylogenetic inference of largely the catalytic domains and on the basis of their protein architectures (Fig. 1). These data show that apicomplexan PDEs belong to at least three major groups or clades. Each clade is widely represented by eukaryotes, in particular, by free-living alveolates, suggesting no PDE family is truly “apicomplexan-specific” as previously inferred (13).

Clade A (orange) is represented most widely by Apicomplexa and also includes human dual-specific (hPDE3) or cAMP-specific (hPDE4,7,8) PDEs (Fig. 1). Most apicomplexan PDEs in clade A resemble hPDE3, having six transmembrane motifs positioned between the N-terminus and catalytic domain. Curiously, clade B (green) includes human PDE1, which is dual-specific, and representatives from free-living alveolates, but only representatives from coccidians are found among the apicomplexans included in the analysis (Fig. 1). The partial conservation of clade B among apicomplexans suggests that these PDEs were dispensable or possibly detrimental to most apicomplexans. Clade B PDEs in *Toxoplasma* (TgPDE4 and TgPDE16) are only expressed in cat stages (22), suggesting that PDEs may be selected during host adaptation. Clade C (purple) is represented by sequences from coccidia and cryptosporidia and also includes human hPDE2,5,6,9,10, and 11, which are either dual-specific (hPDE2,10,11) or cGMP-specific (hPDE5,6,9) (Fig. 1). Given the variety in numbers, diversity of families, and a relatively small portion of alignable residues the nucleotide-binding pocket represents, inference of substrate preference based on the phylogenetic position of apicomplexan PDEs is cautioned. It is quite possible that specificities diverge over time and repertoires of apicomplexan PDEs satisfy distinct requirements of cyclic nucleotide turnover in various parasite life stages and subcellular compartments. We favor a model where the common ancestor of apicomplexans had a large PDE repertoire, similar to free-living alveolates, and PDEs were reduced as apicomplexans evolved smaller and smaller genomes through parasitism. However, it appears that coccidians have also expanded their repertoire of PDEs from a core subset of alveolate PDEs. PDE retention or expansion may offer a certain degree of fortification through functional redundancy, ensuring cyclic nucleotide levels are always tightly regulated.

Structurally, Class I PDEs have an N-terminal regulatory domain and a C-terminal catalytic domain (23). Apicomplexan PDEs have retained this architecture but their regulatory domains have not been defined or characterized (Fig. 1). Notably, one or more transmembrane domains precede the catalytic domain in most apicomplexan PDEs, similar to mammalian PDE3. The prototypical regulatory domains found in Class I PDEs are largely absent or diverged beyond recognition in apicomplexan PDEs. The regulatory domains that are absent in apicomplexan PDEs include upstream conserved region, Per-ARNT-Sim, response regulator receiver, and calmodulin-binding domain (23). Several families of Class I PDEs contain dual GAF domains (named for cGMP-binding PDEs, *Anabaena* adenylyl cyclase, and *Escherichia coli* FhlA), which mediate dimerization and/or allosteric cGMP or cAMP binding, which may regulate catalytic site function (23). A single GAF or GAF-like domain is present in cAMP-specific PDE2 in *Toxoplasma*, which is also conserved in other coccidians. However, the function of the GAF domain has not yet been evaluated in apicomplexans. Due to the general paucity of known regulatory domains in apicomplexan PDEs, it is reasonable to assume that they are regulated by expression and kinase phosphorylation (14). Phosphoproteomics studies have revealed phosphorylation sites on representative apicomplexan PDEs, but their significance awaits further investigation.

Most of what is known about the expression, function, and regulation of apicomplexan PDEs comes from the major human pathogens *Plasmodium*, *Toxoplasma*, *Cryptosporidium*, and *Babesia*. Here, we will focus on these organisms to discuss, compare, and contrast apicomplexan PDE biology.

## CYCLIC NUCLEOTIDE SIGNALING IN *PLASMODIUM*

Functional analyses of *Plasmodium* PDEs have mainly relied on reverse genetics and/or pharmacological approaches. In particular, multiple PDE inhibitors have been extremely valuable in assessing the role of cyclic nucleotide hydrolysis at various developmental stages.

### Asexual blood stages

Malaria pathology is linked to the proliferation of asexual blood stages. Waves of fever arise from the synchronized egress of merozoites from erythrocytes, an event that must be followed by the invasion of fresh red blood cells for the asexual replicative cycle to continue. Precise timing of egress is crucial for parasite survival as premature or late egress leads to non-invasive merozoites (24). The control of both cAMP and cGMP levels by PDEs is central to the tight coordination of egress and invasion. Most of the studies on *Plasmodium* PDEs have focused on the human malaria parasite *Plasmodium falciparum* (Pf) or on the parasites infecting rodents, *Plasmodium berghei* (Pb) and *Plasmodium yoelii* (Py).

#### PDEβ is the only cAMP PDE essential for asexual blood stage by regulating invasion and subsequent development

The *P. berghei* PlasmoGEM global gene knockout project and a *P. falciparum* global transposon mutagenesis project defined *PbPDE*β and *PfPDE*β as the only PDE genes likely essential for asexual blood stages based on an extremely low relative growth rate of gene knockout parasites (25) and the absence of transposon insertion (26), respectively. The expression of *PfPDE*β peaks at late trophozoite and schizont stages. The polytopic enzyme is transported via the endoplasmic reticulum to an apical location, presumably a secretory apical organelle, and then is subsequently discharged to the plasma membrane of individual merozoites within mature schizonts (27).

**Fig 2:**
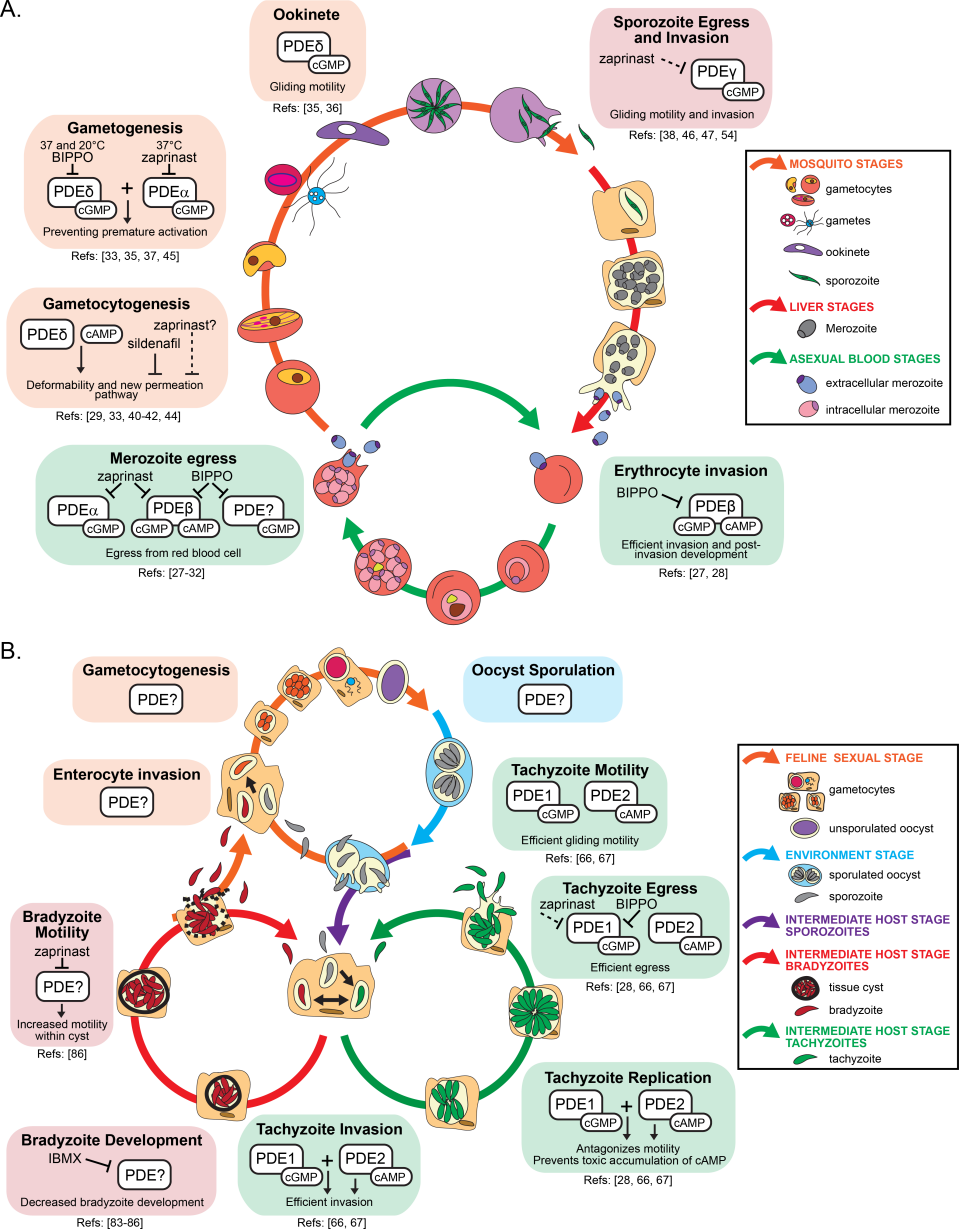
Overview of PDE function in the lifecycles of *Plasmodium* and *Toxoplasma*. The role of PDEs across different life stages in the lifecycles of the apicomplexan parasites: (**A**) *Plasmodium* and (**B**) *Toxoplasma*. (**A and B**) The colored arrows correspond to a different life stage for each parasite. On top of the arrows are depictions of the parasite throughout that specific life stage. The colored boxes correspond to known or suspected effects of PDE inhibitors or PDE activity at specific times during the lifecycle.

Conditional deletion of *PfPDE*β using a DiCre approach led to a dramatic reduction in invasion and rapid post-invasion death, but no defect of egress from the host erythrocyte was reported. Treatment of early ring stages with the PDE inhibitor BIPPO (5-benzyl-3-isopropyl-1H-pyrazolo[4,3-d]pyrimidin-7(6H)-one), a PDE inhibitor derived from a human PDE9 series, phenocopied the PfPDEβ-null post-invasion phenotype suggesting that BIPPO targets PfPDEβ (Fig. 2A). *PfPDE*β conditional deletion led to a dramatic reduction in schizont cAMP and cGMP hydrolytic activity (27). Consistent with these observations, analysis of immunoprecipitated PDEβ from schizonts showed that the enzyme is dual-specific and capable of hydrolyzing both cAMP and cGMP (27, 28). Consistent with this observation, treatment of immunoprecipitated PDEβ with BIPPO abolished both cAMP and cGMP hydrolytic activity confirming that PDEβ is targeted by this molecule (28). Similarly, zaprinast (5-(2-propoxyphenyl)−2,3-dihydrotriazolo[4,5-d]pyrimidin-7-one), a precursor to the human PDE5 inhibitors widely used to trigger cGMP-dependent merozoite egress, has also been shown to be a highly effective inhibitor of cAMP activity as well (29), indicating that it also targets PDEβ (Fig. 2A). However, *PfPDE*β deletion only led to elevated cellular cAMP levels and untimely activation of PKA but not PKG (27). The unchanged cGMP levels are consistent with the absence of an egress phenotype upon *PDE*β deletion, as egress depends on PKG activation, suggesting that at least one other PDE degrading cGMP is active in schizonts to prevent premature egress in the absence of *PDE*β.

Collectively, these data suggest that *Plasmodium* PDEβ shows dual cAMP and cGMP activity. It is the only essential PDE in the clinically relevant asexual blood stages due to its requirement in controlling cAMP levels during the invasion and the immediately following developmental stages. While there is no other PDE capable of hydrolyzing cAMP in schizonts, this developmental stage likely expresses other active cGMP-specific PDEs that likely complement the loss of cGMP hydrolytic activity upon *PDE*β deletion.

#### Epistasis between multiple PDEs is key to time egress by regulating cGMP homeostasis

Treatment with zaprinast induces merozoite egress together with a rapid rise in schizont cGMP levels (24, 27). As conditional deletion of *PDE*β did neither impact schizont egress nor global cGMP levels despite its cGMP hydrolytic activity, this observation indicates that at least another cGMP-hydrolyzing PDE is active in schizont. Conversely, BIPPO also triggers egress in a cGMP-dependent manner (30, 31) indicating that this molecule targets at least a second cGMP PDE in addition to PDEβ. This raises the question as to which additional PDE(s) is responsible for cGMP hydrolytic activity to control merozoite egress.

While *PDE*α, *PDE*γ, and *PDE*δ all show a peak of transcription at the late trophozoite and schizont stages, reverse genetic approaches have demonstrated that PDEα, PDEγ, and PDEδ are each not essential for blood stage replication in different *Plasmodium* species. Clonal PfPDEα-KO and PfPDEδ-KO lines have been generated in *P. falciparum* (32–34), as have PbPDEα-KO, PbPDEγ-KO, and PbPDEδ-KO clonal lines in *P. berghei* (35, 36). Recently, a dual KO of PbPDEα and PbPDEδ was generated, but no obvious growth defect was reported in asexual blood stages (37). Analysis of the expression of PyPDEγ using an epitope-tagged *P. yoelii* transgenic line showed low-level expression in blood stages. However, *PyPDE*γ deletion partially affected blood-stage growth with a twofold decrease in the peak of parasitemia (38), but the exact developmental stage impacted by this deletion remains unknown. In addition to the absence of described roles of non-essential PDEs at the schizont stage, little is known about their localization. All four *Plasmodium* PDEs are integral membrane proteins, and PDE activity in asexual blood stages has been primarily associated with membrane fractions (34). PDEγ was shown to partially overlap with the endoplasmic reticulum marker BiP in *P. yoelii* schizonts (38).

While reverse genetics in *P. yoelii* points to a role of PDEγ in asexual blood stages, multiple lines of evidence indicate that PDEα is involved in schizont egress regulation via cGMP hydrolysis. The relative expression level of *PfPDE*α in schizont is higher than PfPDEγ and PfPDEδ. Recombinant PfPDEα is sensitive to zaprinast and overall cGMP hydrolytic activity was reduced in a *PfPDE*α-KO strain by about 20%, while no effect on cAMP hydrolysis was observed indicating that PDEα is not likely dual specific (32) (Fig. 2A). *In vitro* analysis of the C-terminal domain of PfPDEα in *Escherichia coli* confirmed specific hydrolysis of cGMP (32, 34). PDEα-KO schizonts are still sensitive to zaprinast further confirming that at least one other cGMP-degrading PDE, including PDEβ, is active. However, BIPPO was shown to elevate calcium levels prior to egress in a cGMP-dependent manner (27, 31) but did not seem to target PDEα, at least in *P. berghei* gametocytes, suggesting that a third PDE in addition to PDEα and PDEβ may be active in schizonts (Fig. 2A). Consistent with this hypothesis, both PDEγ and PDEδ have been associated with cGMP hydrolytic activity (33, 38), and PDEδ was found to be the main target of BIPPO in *P. berghei* gametocytes (37).

### Sexual blood stages

Transmission from humans to mosquito is mediated by an obligatory sexual lifecycle phase. Differentiation from asexually replicating stages into non-dividing male and female gametocytes takes place inside red blood cells. During their maturation, *P. falciparum* gametocytes remodel the structural and mechanical properties of the membrane of their erythrocyte host. Some of these modifications play a key role in gametocyte maturation, sequestration in internal organs, subsequent release in the bloodstream, and ability to persist in circulation. Following this period of maturation, the sexual precursors are ready to initiate transmission when ingested by a mosquito in response to environmental signals. At least two distinct roles have been described for cyclic nucleotide monophosphate (cNMP)-dependent signaling during gametocytogenesis, including for the modification of the host erythrocyte and the prevention of premature activation of gametocytes in the mammalian host. As observed for asexual blood stages, transcriptional studies indicate that, despite stage-specific patterns of expression (39), all four PDEs are transcribed in gametocytes with a higher relative expression of PDEδ. This suggests that epistatic interactions are also at play in regulating the biology of gametocytes.

#### cAMP hydrolytic activity is important for gametocyte maturation

*P. falciparum* gametocytes modify the mechanical properties of their erythrocyte host to persist for several weeks in the blood circulation and to be available for mosquitoes. PKA activity was shown to contribute to the stiffness of immature gametocyte-infected erythrocytes (GIE). Rising expression of PfPDEδ in mature gametocyte stages is correlated with a drop in cAMP levels and an increase in infected erythrocyte deformability (29, 40), raising the possibility that PfPDEδ displays some cAMP hydrolytic activity, which was further suggested by the characterization of the enzymatic activity of the recombinant catalytic domain of PfPDEδ (41) (Fig. 2A). Consistent with these observations, treatment with sildenafil or Tadalafil, two PDE inhibitors of unknown specificities in *Plasmodium*, increased cAMP concentration in mature GIE and impaired their circulation in an *in vitro* model for splenic retention (29), induced their retention in the spleen, and impaired the circulation of mature GIE in humanized mice (42), respectively (Fig. 2A). In line with these observations, treatment of *P. berghei*-infected mice with sildenafil increased gametocyte homing to sequestration sites, suggesting that PDE inhibitors may cause gametocytes to become mechanically trapped in the bone marrow and splenic cords (43).

To ensure the transport of nutrients necessary for their survival, *P. falciparum* parasites increase erythrocyte permeability to diverse solutes using the new permeation pathways (NPPs). NPPs were also shown to be regulated by a cAMP signaling cascade (44). The decrease in cAMP levels in mature stages is associated with a slowdown of NPP activity. Consistent with this association, pharmacological elevation of cAMP levels or deletion of *PfPDE*δ resulted in a drastic increase in the permeability of mature GIEs (Fig. 2A).

However, the exact roles of PDEδ and cAMP during gametocytogenesis remain unclear as another study reported consistently low cAMP-PDE activity in gametocytes that were indistinguishable between wild-type and PfPDEδ-KO parasites (33). In addition, gametocyte homing to sequestration sites was not reported in independent transgenic lines lacking Pb*PDE*δ (35–37), raising the possibility of off-target effects of sildenafil or tadalafil or an unexplored role of PDEβ during gametocytogenesis.

#### cGMP degradation by multiple PDEs prevents gametocyte premature activation

The artificial elevation of cGMP levels in mature *P. falciparum* gametocytes by zaprinast at a permissive temperature leads to the formation of fertile gametes (33, 45) indicating that one or more PDEs are active prior to the activation of mature gametocytes. Interestingly, this also suggests that the resulting sustained and high cGMP levels are not detrimental to gamete formation (45), further pointing to a requirement of PDE activity prior to gametocyte activation. A PfPDEδ-KO line showed impaired rounding up and egress associated with decreased cGMP-PDE activity during gametocyte development (33). In parallel, cAMP-PDE activity levels were consistently low and indistinguishable between wild-type and PfPDEδ-KO parasites. It was, therefore, proposed that elevated cGMP levels at a non-permissive temperature in this PDEδ mutant lead to premature activation of gametocytes and defective gametogenesis (33).

Interestingly, mutants lacking *PDE*α or *PDE*δ in *P. berghei* produced fertile gametes (35, 37). However, PbPDEδ-KO gametocytes were less sensitive to BIPPO at both 37°C and 20°C indicating that PDEδ is a target of BIPPO and active at both temperatures in gametocytes (Fig. 2A). Similarly, PbPDEα-KO gametocytes were less sensitive to zaprinast but only at 37°C, confirming that PbPDEα is a target of zaprinast and further showing that PDEα is active in gametocytes at 37°C (37) (Fig. 2A). An epistatic interaction between PbPDEα and PbPDEδ was formally confirmed, as a double PbPDEα/PbPDEδ-KO line showed elevated cGMP levels in non-activated gametocytes and did not produce fertile gametes (37). Such epistasis is likely conserved in *P. falciparum*, as *P. falciparum* PDEδ mutant gametocytes were more sensitive to zaprinast suggesting that a least one other zaprinast-sensitive PDE is active in gametocytes and partially overlaps with requirements for PfPDEδ (33). PDEγ mutants in *P. berghei* and *P. falciparum* did not show any defect in gametocyte formation nor male gamete formation, but it remains possible that either PDEγ or PDEβ are also important, in addition to PDEα and PDEδ, to lower cGMP levels and prevent premature activation of gametocytes.

### Mosquito stages

Gliding motility of malaria parasites is required for mosquito and mammalian host infection. Despite their different morphologies, all invasive lifecycle stages of the malaria parasite—merozoite, ookinete, and sporozoite—rely on a conserved gliding motility machinery to traverse diverse host cell barriers. A role for cGMP degradation by PDEs has been shown to regulate the gliding motility of ookinete and sporozoite. The ookinete is a banana-shaped cell that is formed from a fertilized female gamete in the mosquito midgut. The motile ookinete crosses the blood meal, the mosquito peritrophic matrix, and the midgut epithelium to transform into a sessile oocyst. Sporozoites are then formed and display a slender shape. Sporozoites move significantly faster than ookinetes and migrate from the hemocoel through the salivary gland epithelium into the gland lumen as well as through the skin and across the blood vessel endothelium to ultimately invade hepatocytes.

Sustained ookinete and sporozoite motility requires tight regulation of cGMP levels. Deletion of *PbPDE*δ resulted in a cGMP-dependent marked increase in average gliding speed ookinete (36) and subsequent rounding up of the cells associated with defective invasion of the mosquito midgut (35) (Fig. 2A). These effects were reversed by the inhibition of PKG further confirming the cGMP hydrolytic activity of PbPDEδ (35). Interestingly, PDEβ expression is also high in ookinete, and it remains unknown whether it could also be involved in regulating the colonization of the mosquito gut via degradation of cAMP or cGMP. Deletion of *PyPDE*γ leads to a marked increase in cGMP levels associated with immotile and non-infective sporozoites (Fig. 2A). Diminution of the numbers of motile sporozoites by zaprinast further points to an important role of cGMP signaling to regulate motility and raises the possibility that either PDEγ is targeted by zaprinast or that an additional cGMP-PDE is active in sporozoites (38) (Fig. 2A). Pharmacological and genetic approaches additionally suggested a role of cAMP for the control of gliding motility in sporozoites (46, 47), but the underlying PDE(s) remain(s) to be identified.

Interestingly, elevation of cGMP levels in ookinete and sporozoite led to opposite motility defects, suggesting that different mechanisms of gliding regulation are at play during these two distinct stages. Given the importance of cNMP signaling in regulating the motility of both ookinete and sporozoite, it is possible that such a requirement for PDE is at play to regulate the short-lived motility of merozoite (48) between host cell egress and invasion as PKG was additionally shown to be important for erythrocyte invasion by merozoite (49).

### Liver stages

Multiple requirements for PDEs have been mapped in the blood and mosquito stages, but little is known about their role during the liver stages. While PKAc was proposed to be dispensable for the preerythrocytic stages of *P. berghei* (50), PKG is essential during these developmental stages by controlling merosome release (51, 52). This suggests additional requirements for PDEs during the liver stages. In support of this hypothesis, zaprinast has been shown to rescue depletion of CDPK1 or CDPK5 downstream of PKG activation, indicative of cGMP hydrolytic activity in late liver stages (53). Interestingly, zaprinast was also shown to affect critical processes that occur only between 0 and 3 h following the invasion of hepatocytes by *P. berghei* sporozoites (54), raising the possibility of additional requirements of cNMP signaling in early liver stages. The growing panels of conditional or stage-specific genetic systems will likely be instrumental in identifying the PDEs regulating these processes.

### Current challenges in studying *Plasmodium* PDEs

#### Spatiotemporal regulation of cNMP signaling by PDEs

While general requirements for PDEs have been mapped across most of the *Plasmodium* lifecycle, little is known about their exact spatiotemporal regulation. The measure of cNMP levels has so far relied on biochemical approaches on parasite populations by necessity but does not account for the central role of local and temporal control of such dynamic and diffusible signaling molecules. Multiple robust and sensitive Förster resonance energy transfer (FRET)-based cNMP sensors have recently been developed in eukaryotic systems. This includes two cGMP probes based on the carboxyl-terminal cyclic nucleotide-binding domain of *P. falciparum* PKG (55) that could likely be implemented in *Plasmodium*. In parallel to direct cNMP imaging, a refinement of the localization of PDEs together with related cyclase and effectors will allow us to better understand the spatiotemporal regulation of cNMP signaling. New accessible super-resolution imaging approaches have been recently implemented in *Plasmodium*. This includes ultrastructure expansion microscopy (56) that together with specific markers or general protein or membrane dyes (57) may allow us to refine the geography of cNMP signaling across developmental stages.

#### Regulation of *Plasmodium* PDEs

Regulation of PDEs’ hydrolytic activity is important for the modulation of cellular functions. Multiple mechanisms have been involved in the regulation of PDE functions such as localization, protein-protein interaction, or post-translational modifications (PTMs) (58). For example, the compartmentalization of cyclic nucleotide signals by tethering PDEs to a precise location via binding partners has been well documented. It is worth mentioning here that, in *Plasmodium*, no PDE interactors have been described so far and, apart from PDEβ apical localization in merozoite (27), little is known about PDE localization across the various stages of the *Plasmodium* lifecycle. PTMs also directly regulate PDE activity and location. For example, in human cells, PKA or PKG phosphorylation of PDE3, PDE4, PDE5, and PDE8 directly stimulates catalytic activity. Phosphorylation can also indirectly regulate the function of PDE by affecting their localization. In rats, phosphorylation of PDE10A2 by PKA attenuates its palmitoylation and associated membrane localization is lost (59). Finally, the ubiquitination of PDEs by SCF E3 ligases has also been shown to be important to regulate proteostasis or interaction with protein partners in other eukaryotes (60, 61). Proteomic approaches mapped phosphorylation sites on all four PDEs across various stages, including asexual blood stages (PDEα and PDEβ), gametocytes (PDEα and PDEβ), ookinetes (PDEδ), and sporozoites (PDEγ). Interestingly, in *P. berghei* ookinete, PDEδ S310 was shown to be phosphorylated in a PKG-dependent manner raising the possibility that its function could also be regulated in a PKG-dependent feedback loop. Recently, ubiquitination of PDEβ was reported in *P. falciparum* asexual blood stages (62), but its functional relevance remains to be elucidated.

## CYCLIC NUCLEOTIDE SIGNALING IN *TOXOPLASMA*

As described for *Plasmodium*, cyclic nucleotide signaling is also critical in *Toxoplasma* (63, 64). With roles in motility and development, cyclic nucleotide signaling is integral to multiple life stages of *Toxoplasma*. Here, we will discuss the role of PDEs in tachyzoites, bradyzoites, merozoites, and sporozoites.

### Conservation, expression, and substrate specificities of *Toxoplasma* PDEs

Despite possessing only five nucleotide cyclases, *Toxoplasma* encodes 18 PDEs (13), which is a relatively high number compared to *Plasmodium*, *Cryptosporidium*, or *Babesia*, which have between two and four PDEs each. Phylogenetic analysis indicates that *Toxoplasma* retained a subset of PDEs from their free-living alveolate ancestors, which were subsequently replicated (e.g., clade A) in a common ancestor of coccidians (Fig. 1). Among the coccidians with annotated genomes, members of the Sarcocystidae family generally encode orthologs of the 18 *Toxoplasma* PDEs. *Neospora*, *Hammondia*, *Besnoitia*, and *Cystoisospora* encode a 19th PDE that is absent in *Toxoplasma*, but *Cystoisospora* lacks an ortholog of TgPDE15, bringing its total to 18. *Sarcocystis* appears to be an outlier in Sarcocystidae, encoding only 11 PDEs. The genome of *Sarcocystis* is roughly twice the size of *Toxoplasma*’s due to larger introns and intergenic sequences but has approximately 20% fewer genes, which may partially explain its PDE reduction. Two sequenced representatives of the Eimeriidae family, *Eimeria* and *Cyclospora*, encode 13 PDEs each. Interestingly, the two most important PDEs to *Toxoplasma*, TgPDE1 and TgPDE2, share syntenic orthologs in all coccidian parasites. Additional studies are needed to define the function of PDEs in other coccidian parasites.

*Toxoplasma* PDEs are not equally expressed across all life stages based on transcript abundance, protein abundance, detection of epitope-tagged PDE fusions, and functional analyses (22, 65, 66). Each life stage appears to express a unique subset of PDEs with some overlap of subset members. In tachyzoites, at least 11 PDEs are detectable at the protein level but only TgPDE1 and TgPDE2 substantially contribute to tachyzoite fitness (66, 67). TgPDE1 and TgPDE2 regulate motility, invasion, replication, and egress in the tachyzoite lytic cycle (66, 67) but may also serve similar or unique functions in other life stages (Fig. 2B). While studies examining bradyzoite, merozoite, gametocyte, and sporozoite stage PDEs are needed to further elucidate the role of PDE life-stage specificity, there are many new studies uncovering the role of PDEs in tachyzoites.

Efforts have been made to predict PDE substrate specificity *in silico* with modest success (13). Similarly, attempts at defining PDE substrate preference using recombinantly expressed soluble PDE catalytic domain fusions were inconclusive due to weak or absent PDE activity (66). To date, only immunoprecipitation of endogenously expressed epitope-tagged PDEs from tachyzoites has been used successfully to define PDE substrate preference *in vitro* (13, 28, 66, 67). Although these preparations are not pure, liquid chromatography with tandem mass spectrometry did not detect off-target PDE capture (66, 67). Dual-specific PDEs include TgPDE1, 5, 6, 7, 8, 9, 10, 12, 13, and 18 (13, 28, 66, 67). However, it is crucial to consider that these dual-specific PDEs may primarily degrade cGMP or cAMP in *Toxoplasma*. For example, TgPDE1, 7, and 9 prefer cGMP to cAMP as a substrate (28, 67). The only known cAMP-specific PDE in *Toxoplasma* is TgPDE2 (28, 66, 67). In addition to showing cAMP activity, knockdown of TgPDE2 elevated cAMP levels in tachyzoites (28). Unlike *Plasmodium*, *Toxoplasma* does not appear to possess a cGMP-specific PDE, although TgPDE3, 4, 11, 14, 15, 16, and 17 have not yet been tested. These results should be validated by measuring cyclic nucleotide levels following inhibition or knockdown of each TgPDE as has been demonstrated for TgPDE2 (28).

### Tachyzoites

While cyclic nucleotide signaling enzymes are expressed in every life stage of *Toxoplasma*, the bulk of knowledge concerning cGMP and cAMP signaling comes from tachyzoite research. Cyclic GMP activation of TgPKG is required for tachyzoite motility to permit extracellular migration, host cell invasion, and host cell egress (24, 31, 68–72). TgPKG regulates motility by controlling microneme secretion. TgPKG promotes microneme secretion by mobilizing Ca^2+^ (70, 71) for TgCDPK activation (e.g., TgCDPK1 and TgCDPK2A) (73, 74) and by mechanism(s) that cannot be bypassed by Ca^2+^ (72). It is unclear whether TgPKG also controls steps downstream of microneme secretion such as surface microneme protein translocation by the actin-myosin motor or microneme protein shedding by rhomboid proteases. Additional studies are needed to identify the relevant substrates of TgPKG required for Ca^2+^ mobilization, microneme secretion, and motility. Conversely, cAMP binds the protein kinase A regulatory subunit (TgPKAr) to release and activate the protein kinase A catalytic subunit (TgPKAc). Following the invasion, TgPKAc1 activation suppresses motility to prevent premature egress and promote intracellular replication (75, 76). The mechanism by which TgPKAc1 suppresses motility is not fully understood but appears to involve recalibrating Ca^2+^ levels following invasion (76). Therefore, cGMP and cAMP levels must be tightly controlled to facilitate intracellular replication and cell-to-cell transmission. While some cyclic nucleotide signaling is conserved across Apicomplexa, it is important to remember that PKA is essential for *Plasmodium* invasion, but not *Toxoplasma* invasion. Instead, both *Plasmodium* and *Toxoplasma* require PKG signaling for motile processes.

#### TgPDE1 cooperates with at least one other PDE to regulate cGMP levels in tachyzoites

The initial evidence for cGMP turnover in tachyzoites stemmed from inhibitor studies. Inhibitors of cGMP-PDEs, such as zaprinast or BIPPO, elevate cGMP and activate TgPKG-dependent Ca^2+^ flux, microneme secretion, and motility (28, 30, 69–71). Prolonged treatment with zaprinast or BIPPO blocks tachyzoite plaque formation, suggesting that sustained cGMP elevation is toxic (30, 66, 71). To identify the targets of zaprinast and BIPPO, selected PDEs were immunoprecipitated and tested for activity in the presence of these inhibitors (28, 67). Zaprinast and BIPPO inhibited TgPDE1 and TgPDE9 (28, 67), with BIPPO also inhibiting TgPDE7 (67). These data indicate that multiple PDEs cooperate to regulate cGMP levels in tachyzoites, but additional approaches are necessary to delineate their individual spatiotemporal roles.

Initial attempts to knock out TgPDE1 in tachyzoites were unsuccessful (75). Similarly, a genome-wide CRISPR disruption screen indicated that TgPDE1 substantially contributes to tachyzoite fitness (65). Conditional loss of TgPDE1 clearly demonstrated its importance for tachyzoite lytic lifecycle growth based on plaquing defects (28, 66, 67). Surprisingly, TgPDE1 was individually dispensable for invasion, replication, and egress but important in the parasite’s extracellular migration and microneme production or secretion (66). Combinatorial loss of TgPDE1 and TgPDE2 impaired tachyzoite motility, invasion, replication, egress, and growth to a greater extent than the loss of either PDE separately (67). However, it is unlikely that TgPDE1 and TgPDE2 cooperate to control cGMP levels since TgPDE2 is cAMP-specific (28, 66, 67). Instead, combinatorial loss of TgPDE1 and TgPDE2 likely produces a gain of function defects from the simultaneous elevation of cGMP and cAMP. Therefore, other cGMP-hydrolyzing PDEs may compensate for the loss of TgPDE1. These cGMP-focused PDEs may form cGMP-hydrolyzing factories at different microdomains within the parasites to be able to respond to cell signaling at different places within the parasite.

In tachyzoites, TgPDE1, TgPDE7, TgPDE8, and TgPDE9 localize to the plasma membrane placing them in close proximity to cGMP production and PKG signaling (13, 66, 75, 77). TgPDE7 is the most highly expressed PDE in tachyzoites (13, 66) but is dispensable based on a genome-wide CRISPR screen and conditional knockdown (65, 66). This may be due to incomplete knockdown or compensation by TgPDE1. TgPDE8 and TgPDE9 are also dispensable in tachyzoites (13, 66). TgPDE9 accumulates at the apical pole of the plasma membrane as described for TgGC (13, 28, 66, 67). A conditional knockdown of TgPDE9 revealed that it has a modest effect on the average size of plaques formed, while the number of plaques was not affected (66). Additional studies are needed to identify the exact combination of PDEs that control cGMP levels in tachyzoites.

#### TgPDE2 regulates cAMP levels in tachyzoites

Attempts to knock out *TgPDE2* individually (75), or as part of a CRISPR disruption screen (65), suggested that *TgPDE2* is important for tachyzoite growth and/or motility. In agreement, conditional loss of TgPDE2 severely reduces tachyzoite plaque size, confirming that it is a primary regulator of the lytic lifecycle (28, 66, 67). There is evidence that TgPDE2 regulates several steps of the lytic lifecycle including attachment, invasion, replication, microneme secretion, and extracellular migration (66) (Fig. 2B). Loss of TgPDE2 also partially antagonized tachyzoites’ ability to egress in response to Ca^2+^ or cGMP agonists (28, 66, 67). Therefore, the small plaque phenotype caused by the loss of TgPDE2 is likely due to the sum of several defective processes throughout the lytic cycle.

The mechanism by which TgPDE2 controls the tachyzoite lytic lifecycle likely involves cAMP hydrolysis. To date, TgPDE2 is the only known apicomplexan PDE that exclusively hydrolyzes cAMP (28, 66, 67). Conditional loss of TgPDE2 elevates cAMP in tachyzoites (28), likely leading to hyperactivation of TgPKAc1. Similar to TgPDE2 depletion, overexpression of TgPKAc1 blocks tachyzoite replication (75). Conversely, loss of TgPKAc1 or overexpression of TgPKAr causes premature egress or restless invasion (75, 76). Taken together, cAMP levels must be tightly controlled by TgPDE2 to facilitate TgPKAc1 activation to prevent premature egress but also prevent TgPKAc1 hyperactivation for replication.

#### Spatiotemporal regulation of cNMP signaling by PDEs in tachyzoites

While more studies will be needed to elucidate the precise roles of PDEs in the tachyzoite lytic lifecycle, there is sufficient data to propose a spatiotemporal model of PDE function to test. For motility, enough cGMP must accumulate at the plasma membrane to activate the plasma membrane-bound isoform of TgPKG (72). This involves converting GTP to cGMP by TgGC at the apical plasma membrane (78, 79) while inactivating PDEs that degrade cGMP such as TgPDE1. Activation of TgPKG likely induces the phosphorylation of a network of proteins for Ca^2+^ influx or mobilization, microneme secretion, and glideosome activation (24, 31, 68–72). Thus, cGMP signaling is required for host cell egress, extracellular migration, and host cell invasion. Following invasion, cGMP synthesis is inhibited, while cGMP pools are hydrolyzed by cGMP-PDEs to prevent further activation of TgPKG. At the same time, ATP is converted to cAMP by TgACs to activate TgPKAc1 at the plasma membrane. Once activated, TgPKAc1 diffuses away from TgPKAr to turn off motility and promote replication (75, 76). Potential substrates of PKA have been identified through phosphoproteomics but have not been functionally investigated (75). Following replication, cAMP synthesis is likely reduced, and the remaining cAMP pools are degraded by TgPDE2. TgPDE2 has a punctate cytomembranous localization (13, 66, 75), but the significance of its localization remains unknown. It is unclear how TgPDE2 associates with cytomembranes, but its catalytic domain is predicted to be cytosolic. Furthermore, there appears to be significant cross talk between cGMP, cAMP, and Ca^2+^ signaling pathways in tachyzoites via resolved kinase-dependent mechanisms (28, 75). Altogether, PDEs function to regulate the timing and amplitude of tachyzoite motility via cNMP hydrolysis, thereby promoting lytic cell cycle progression.

### Bradyzoites

Immunocompetent individuals are generally able to suppress acute infections with *Toxoplasma* tachyzoites. To persist, tachyzoites disseminate and convert into bradyzoites within tissues such as the brain, eye, and muscle. Bradyzoites are protected from immunity and anti-*Toxoplasma* medications by downregulating tachyzoite antigens, persisting within immune-privileged tissues, sequestration behind blood barriers (e.g., brain and retina), secreting effectors to subvert intracellular immunity, slowing growth and motility, and forming a tissue cyst wall within host cells (80–82). Bradyzoites are also vital for *Toxoplasma* transmission between hosts through the ccarnivorism of raw or undercooked meat (81). Alternatively, *Toxoplasma* infections are transmitted by ingestion of feline-derived oocysts in contaminated food or water (81).

Tachyzoite-to-bradyzoite differentiation is antagonized by prolonged cAMP signaling through TgPKAc3 (83). The PDE inhibitor IBMX (3-isobutyl-1-methylxanthine) elevates cAMP and maintains tachyzoites under bradyzoite induction conditions *in vitro* (83–85). IBMX blocks the activities of immunoprecipitated tachyzoite TgPDE1, TgPDE2, TgPDE7, and TgPDE9 (67) but has little effect on tachyzoite fitness (66). Since TgPDE2 is the only known cAMP-specific PDE in *Toxoplasma*, and most important for tachyzoite fitness, it is the most likely target of IBMX with regard to cAMP elevation for tachyzoite maintenance. Furthermore, loss of TgPKAc3, which is activated by cAMP, induces bradyzoite development and is unresponsive to bradyzoite induction conditions and IBMX (83). Premature bradyzoite development from TgPKAc3 deletion may explain why these mutants form smaller tachyzoite plaques *in vitro* and fewer brain tissue cysts *in vivo*.

Bradyzoites reactivate (excyst) from unknown signals in tissues and either differentiate into virulent tachyzoites or form secondary bradyzoite tissue cysts, depending on host immunocompetence. Bradyzoites must also reactivate following ingestion to infect the intestines of its new host (81). Therefore, motile bradyzoite reactivation is crucial for both *Toxoplasma* virulence and transmission. New evidence indicates that cyclic nucleotide signaling also has a role in bradyzoite reactivation and motility. In tachyzoites, the PDE inhibitor zaprinast elevates cGMP, and subsequently Ca^2+^, stimulating microneme secretion, motility, and egress (69–72). Zaprinast also induces motility in bradyzoites within *in vitro* and *ex vivo* tissue cysts, indicating that one or more PDEs must suppress cGMP signaling to inhibit their motility (86) (Fig. 2B). However, bradyzoites are much less responsive to zaprinast compared to tachyzoites, likely due to a significant reduction of stored Ca^2+^ and ATP (86). Zaprinast-treated bradyzoites fail to reactivate (excyst), indicating additional signals or factors are necessary for cyst wall destruction (86).

Reverse genetic studies are needed to identify PDEs that regulate bradyzoite development and/or reactivation. The PDEs that are most likely to be expressed in bradyzoites are TgPDE5 and TgPDE6 based on transcriptomics (22). Interestingly, many other PDEs see a substantial increase in transcript abundance during the change from tachyzoites to bradyzoites, while some see a slight decrease (22). These changes in transcript abundance help solidify the idea that cyclic nucleotides must be tightly regulated by PDEs in order for an orderly transition from one life stage to another.

### Merozoites and sporozoites

*Toxoplasma* undergoes its sexual lifecycle exclusively in the feline intestines. This unique habitat is rich in linoleic acid, as felines are the only mammals that lack an enzyme required for linoleic acid metabolism (87). Due to the current lack of a robust *in vitro* system to study merozoites, much of our knowledge of merozoites and sporozoites has come from transcriptomic studies of feline feces and enteroepithelial tissue. These studies have demonstrated that TgPDEs tend to be expressed during one or two of the life stages (tachyzoite, bradyzoite, or merozoite) but not all three (22). For example, while TgPDE2 is essential for tachyzoite fitness, TgPDE2 is expressed more in late feline enteric stages (22). This may suggest a role for TgPDE2 regulating cAMP signaling in monitoring the sexual development of new *T. gondii* progeny. TgPDE8 is highly expressed in merozoites, which may explain the trouble in detecting tachyzoite TgPDE8. Other TgPDEs that are most likely expressed in merozoites are TgPDE3, 4, 12, and 14–18 due to the mRNA transcript levels being orders of magnitude higher than their tachyzoite or bradyzoite counterparts (22). Additional studies are needed to identify how merozoite PDEs contribute to the sexual development and genetic variation of *T. gondii* and how merozoite PDEs prepare the parasite to become an invasive sporozoite.

### Current challenges in studying *Toxoplasma* PDEs

#### Functional redundancy of PDEs in *Toxoplasma*

Functional redundancy within the 18-member PDE family in *Toxoplasma* has made it difficult to assess their importance. A screen for essential PDEs in tachyzoites revealed at least four PDEs (TgPDE1, 2, 5, and 9) have non-redundant and/or semi-redundant function in tachyzoites (66). A similar screen performed in bradyzoites, merozoites, and sporozoites would likely reveal unique fitness-conferring PDEs. Furthermore, screening for synthetic lethality using a combinatorial knockout/knockdown or CRISPR multiplexing strategy could reveal essential PDE subsets in each life stage.

#### Regulation of *Toxoplasma* PDEs

Many eukaryotic PDEs are regulated by phosphorylation of their N-terminal domains. Since apicomplexan PDEs lack obvious regulatory domains, it is likely that they are regulated by phosphorylation and other post-translational modifications. Several phosphosites on TgPDE1 are upregulated by treatment with A23187 (Ca^2+^ ionophore) and BIPPO in a CDPK3-dependent manner (28). In addition, zaprinast treatment also increases TgPDE1 phosphorylation (88). TgPDE1 is considered the most important PDE for cGMP in tachyzoites, yet it is capable of degrading certain concentrations of cAMP *in vitro*. It is possible that phosphorylation of TgPDE1, and other dual-specific PDEs like TgPDE7 and TgPDE9, may activate, inhibit, or bias them toward cGMP or cAMP hydrolysis *in vivo*. Likewise, co-immunoprecipitation studies suggest that TgPDE2 interacts with various kinases, indicating that TgPDE2 may also be regulated by phosphorylation (66). In support, TgPDE2 is differentially phosphorylated in response to A23187 (28) or zaprinast (88), suggesting that TgPDE2 is phosphorylated by cGMP- and/or Ca^2+^-dependent kinases. Since TgPDE2 is cAMP-specific, these phosphorylations are more likely to affect its activity than substrate preference. Furthermore, TgPDE2 is hyperphosphorylated in the absence of PKA, indicating that cAMP signaling antagonizes its phosphorylation (75). TgPDE2 also possesses a GAF domain (66). This GAF domain may act as an allosteric activator or inhibitor of PDE hydrolysis activity through cyclic nucleotide binding to the GAF domain. GAF domains have also been shown to promote dimerization in other organisms (89). Whether *Toxoplasma* PDEs are regulated by phosphorylation or cNMP-binding requires further investigation.

## CYCLIC NUCLEOTIDE SIGNALING IN *CRYPTOSPORIDIUM* AND *BABESIA*

Evidence for cyclic nucleotide signaling in Apicomplexa extends beyond *Plasmodium* and *Toxoplasma*. Recent studies of other clinically important parasites, including *Cryptosporidium* and *Babesia*, offer insights into the evolution and adaptation of apicomplexan signaling.

### Cyclic nucleotide signaling in *Cryptosporidium*

*Cryptosporidium* is an intestinal parasite and a leading cause of severe diarrhea in children. Unlike most apicomplexans, *Cryptosporidium* undergoes its asexual and sexual lifecycles in a single host (90). Infections occur through the ingestion of thick-walled oocysts that release motile sporozoites that infect the epithelium of the small intestine. Sporozoites develop into trophozoites, which differentiate into type I meronts (90). Type I meronts contain eight motile merozoites that egress and infect other intestinal epithelial cells (asexual lifecycle) (90). After three replication cycles, merozoites may develop into undifferentiated gamonts (a precursor of sexual stages) (90). Gamonts develop into male (microgamont) or female (macrogamont) stages. Microgamonts release motile microgametes that fertilize macrogamonts to form a zygote (90). Fertilized zygotes produce thick-walled oocysts that are excreted into the environment or thin-walled oocysts that can release motile sporozoites, creating an auto-infection cycle (90). It is currently not known how cyclic nucleotide signaling impacts the development of *Cryptosporidium*. However, sporozoites, merozoites, and presumably microgametes all require motility for invasion, with recent studies implicating cyclic nucleotide signaling as an important regulator of *Cryptosporidium* motility (91–93).

*Cryptosporidium* harbors a complete repertoire of cyclic nucleotide signaling genes. *Cryptosporidium parvum*, a common human and bovine species, encodes three ACs (cgd5_1290, cgd2_1270, and cgd4_3100), one GC (cgd3_1110), three PDEs (cgd3_2320, cgd6_500, and cgd6_4020) (Fig. 1), one PKG (cgd8_750), and one PKA catalytic subunit (cgd3_3040) regulated by a PKA regulatory subunit (cgd7_120). As the three *Cryptosporidium* PDEs have not been named previously, we used our phylogenetic analysis to propose nomenclature to them. Phylogenetic analysis indicates that cgd3_2320 (CpPDE1) shares similar length and domain structure and clades closest with the dual-specific PDE, TgPDE5 (Fig. 1). Cgd6_500 (CpPDE2) has a similar length and domain architecture as TgPDE2 and clades closely with TgPDE2 in our phylogenetic analysis suggesting a possible cAMP specificity (Fig. 1). Interestingly, alignment of CpPDE2 with TgPDE2 revealed that CpPDE2 has remnants of a GAF regulatory domain but has degenerated to the point where it is not identified in conserved domain searches (Fig. 1). This could mean that either the GAF domain in TgPDE2 is not required for PDE activity, that the degenerated GAF in CpPDE2 is still functional, or that CpPDE2 and TgPDE2 are regulated in distinct ways. Cgd6_4020 (CpPDE3) shares a higher sequence identity with TgPDE3 (BLAST). CpPDE3 also clades closely to CpPDE2 and TgPDE2 but is much shorter, has transmembrane domains, and lacks a GAF domain.

A 2019 study by Tandel et al. created transgenic reporter parasite lines followed by RNA sequencing to analyze *C. parvum* gene expression in both asexual and sexual life stages (93). In sporozoites, CpPKG and CpGC transcripts were nearly undetectable, indicating that cGMP signaling is likely dispensable for sporozoite motility. In contrast, CpPKAc and CpPKAr are highly expressed in sporozoites, indicating that cAMP may be an important regulator of *Cryptosporidium* sporozoite motility as described in *Plasmodium* (46, 94). This concept is further supported by the transcript expression of two ACs (cgd5_1290 and cgd4_3100) in sporozoites, albeit at low levels (93). Of the three PDEs, only CpPDE1 is expressed in *Cryptosporidium* sporozoites (93), which is presumed to degrade cAMP due to the lack of CpGC and CpPKG expression in sporozoites. *Cryptosporidium* sporozoites can infect immortalized epithelial cell lines such as HCT-8 (human ileocecal adenocarcinoma) cells and develop for up to 3 days. At 24 h, *C. parvum* in HCT-8 cultures are 100% asexual (e.g., trophozoites, meronts, and merozoites) (93). By 48 h, sexual stages (e.g., female gametes, male gamonts, and male gametes) predominate over asexual stages in the HCT-8 cultures with >80% of the stages being sexual stages (93). In comparison to the initial sporozoite transcriptome, the *Cryptosporidium* transcriptomes at 24 and 48 h in HCT-8 cell cultures show some differences in cyclic nucleotide gene expression. For instance, CpGC and CpPKG are both upregulated in asexual (24 h) and sexual stages (48 h) (93), suggesting a need for cGMP signaling in the development and/or motility of *C. parvum*. A separate study demonstrated that silencing CpPKG expression inhibited merozoite egress at 24 h in HCT-8 cultures (92). Furthermore, the apicomplexan PKG inhibitor known as compound 1 {[trisubstituted pyrrole, 4-[2-(4-fluorophenyl)-5-(1-methylpiperidine-4-yl)-1H-pyrrol-3-yl] pyridine} (95) has an EC_50_ of 13.47 µM against *C. parvum* in HCT-8 cultures (91). CpAC (cgd4_3100), CpPKAc, and CpPKAr are also upregulated in asexual (24 h) and sexual stages (48 h) (93), indicating a need for cAMP in the development and/or motility of *C. parvum*. Two PDEs are upregulated in asexual stages, CpPDE1 and CpPDE2, whereas all three PDEs are expressed in the sexual stages (93). CpPDE1 may be the most critical PDE in *C. parvum* based solely on transcript levels (93). It is currently not known whether any PDE in *Cryptosporidium* is essential, whether they degrade cAMP, cGMP, or both, whether they are inhibited by PDE inhibitors such as IBMX, zaprinast, or BIPPO, or how they are regulated.

### Cyclic nucleotide signaling in *Babesia*

*Babesia* is a tick-borne blood parasite of humans and animals that causes babesiosis, which can be life-threatening in those who are immune-deficient, lack a spleen, or are elderly (96). Ticks ingest gametes from infected animal blood where they fertilize in the tick gut and produce zygotes (97). Zygotes develop into motile unicellular kinetes that migrate to the tick salivary gland and develop into sporozoites for transmission (98). *Babesia* sporozoites are injected into humans during a tick blood meal where they subsequently infect red blood cells (97, 98). In human red blood cells, *Babesia* will develop into asexual stages, cycling between trophozoites and merozoites (97, 98). Merozoite egress from red blood cells causes hemolytic anemia and flu-like symptoms associated with babesiosis (98).

*Babesia microti* is the predominant species of *Babesia* in humans (96). Sequence analysis of the *B. microti* genome, which is the smallest apicomplexan genome sequenced to date, revealed conserved cyclic nucleotide signaling genes. *B. microti* encodes one GC (BMR1_03g00165) with the prototypical P4-ATPase-GCα architecture found in apicomplexans and other protists (99) and one AC (BMR1_04g07620) containing two N-terminal cyclase homology domains, resembling ACβs in other apicomplexans like *Toxoplasma*, *Plasmodium*, and *Cryptosporidium*. To respond to cGMP, *B. microti* encodes one PKG (BMR1_04g05785). For cAMP signaling, *B. microti* encodes two PKA catalytic subunits (PKAc1: BMR1_04g07710; PKAc2: BMR1_03g00225), which are likely inhibited by the PKA regulatory subunit (BMR1_01G03115) when cAMP levels are low or absent. For cyclic nucleotide turnover, *B. microti* encodes two PDEs (BMR1_03g01065 and BMR1_03g02245) as shown in Fig. 1. As the two *B. microti* PDEs have not been named yet, we used our phylogenetic analysis to propose nomenclature to them. BMR1_03g01065 (BmPDEα) is orthologous to PfPDEα and PfPDEδ, but the six transmembrane domains found in the N-terminus of all *Plasmodium* PDEs are missing, indicating that it may regulate cGMP (or cAMP) in the cytosol or associates with membranes in other ways (Fig. 1). BMR1_03g02245 (BmPDEβ) is orthologous to PfPDEβ and PfPDEγ, which are dual- and cGMP-specific, respectively (Fig. 1).

A 2016 gene expression study of human *B. microti* isolates found that 98.3% of the 3,567 protein-coding genes in the *B. microti* genome were expressed in intraerythrocytic blood stages of mice by RNAseq (100). Seven of the eight *B. microti* cyclic nucleotide signaling genes described above were expressed in the second quartile or higher relative to all genes, with BmACβ (BMR1_04g07620) expressed in the third quartile (100). Interestingly, the two *Babesia* BmPDEs showed the highest expression among the eight cyclic nucleotide signaling genes with BmPDEα in the first quartile and BmPDEβ in the top 10% of all genes (100). This expression study suggests that *Babesia* utilizes cGMP and cAMP signaling in intermediate hosts. Whether *Babesia* utilizes cyclic nucleotide signaling in its definitive host awaits further investigation.

There is growing direct evidence for cyclic nucleotide signaling in *Babesia*. The apicomplexan PKG inhibitor compound 1 has an IC_50_ of 2.4 µM against *Babesia bovis* in bovine erythrocyte cultures (101), suggesting cGMP signaling in *Babesia* is essential. A recent study by Elsworth et al. determined that the cGMP-PDE inhibitor BIPPO, and the cell permeable cGMP analog (8-Br-cGMP), induces *Babesia divergens* motility and egress in erythrocyte culture, which could be antagonized by compound 1 (102). Conditional knockdown of BdPKG in *B. divergens* blocked growth and prevented natural and 8-Br-cGMP-induced egress (102). Using chemical genetics, compound 1 and ML10 were shown to target BdPKG. However, at concentrations required for killing *B. divergens*, there were secondary targets contributing to growth inhibition (102). Combinatorial PKG depletion and inhibition with compound 1 demonstrated that PKG was also important for host cell invasion (102). Therefore, the role of cGMP signaling in initiating *Babesia* motility is consistent with *Plasmodium* and *Toxoplasma*. The PKA inhibitor H89 weakly induced *B. divergens* merozoite egress (102), suggesting that BdPKAc1 and/or BdPKAc2 in *Babesia* functions similarly to TgPKAc1 for suppressing egress in *Toxoplasma* (75, 76). Conversely, BdPKA inhibition strongly inhibited invasion of *B. divergens* merozoites (102), suggesting that cAMP signaling is required for *Babesia* invasion, as seen in *Plasmodium* (103, 104), but not *Toxoplasma* (75, 76). The cGMP-PDE inhibitor BIPPO has an IC_50_ of 7.6 µM against *B. divergens* (102), suggesting that at one or both *Babesia* PDEs are essential. It remains unclear which *Babesia* PDE regulates cGMP and cAMP levels, but the *Babesia* orthologs of PfPDEα (cGMP-specific) and PfPDEβ (cAMP > cGMP) may function similarly to their *Plasmodium* counterparts. Further genetic studies are needed to confirm the essentiality of cyclic nucleotide signaling genes beyond PKG in *Babesia* since inhibitors like H89 and BIPPO may have unwanted off-target effects.

## APICOMPLEXAN PDEs AS DRUG TARGETS?

### PDEs are validated drug targets

PDEs are being pursued as therapeutic targets for several human diseases, including those affecting the cardiovascular system, the nervous system, metabolism, fertility, cancer, and immunity (58). The majority of current PDE-targeted therapeutics are competitive blockers of substrate binding at the catalytic site. Such an approach currently lacks the ability to selectively target a specific isozyme within a single PDE family. However, novel strategies to target PDEs are emerging and include enhancing catalytic activity, altering compartmentalization, or modulating post-translational modifications (58).

This is clearly an exciting time in the apicomplexan PDE field, but much work remains to be done to assess their therapeutic potential as drug targets for apicomplexan diseases. Multiple small molecules have already been shown to target apicomplexan PDEs with antiproliferative activity including BIPPO and zaprinast. While their exact specificities on apicomplexan PDEs remain to be comprehensively characterized for each parasite stage, these molecules provide proof of the principle that multiple PDEs can be simultaneously targeted across various lifecycle stages in different parasites.

### PDE inhibition shows multistage activity with a timing activity to be refined

Cyclic GMP signaling has long been explored as a potential target for new anti-apicomplexan drugs. This pathway is essential for many of the key developmental stages of the parasite lifecycle (10, 63), giving hope that targeting cGMP signaling might give rise to drugs that treat the diseases, block their transmission, and prevent the establishment of infection. With our current knowledge about the multistage requirements of PDEs, it is likely that drugs targeting these enzymes will address some of the desired characteristics for new drugs in their target candidate profiles. For example, this includes molecules with activity against *Plasmodium* asexual blood-stage parasites and hepatic schizonts, as well as molecules that block transmission by targeting parasite gametocytes (105).

So far, cGMP signaling as a drug target has mainly been explored via the inhibition of the only known cGMP effector, PKG. In the early 2000s, researchers at Merck developed the first inhibitors of apicomplexan PKGs commonly known as compound 1 (106) and compound 2 (107). These compounds were shown to block apicomplexan infectivity through the inhibition of PKG but were triaged from further drug development due to secondary targets (108, 109) and genotoxicity (107). More recently, PKG inhibitors with greater potency, selectivity, and improved cytotoxicity profiles have been generated and characterized. For example, ML10 has an IC_50_ value of 160 pM against recombinant *P. falciparum* PKG, an EC_50_ value of 2.0 nM in 72-h blood stage growth inhibition assays, and a clean *in vitro* cytotoxicity profile against multiple mammalian cell lines (110–112). It also shows transmission-blocking activity with an EC_50_ of 41.3 nM in a standard membrane-feeding assay, which measures the ability of an inhibitor to block the development of oocysts in mosquitos. The inhibitory activity likely encompassed the essential requirements for PKG in the activation of gametogenesis and in ookinete motility. Finally, drug resistance is not readily generated to this class of inhibitor (110). Based on its drug potential in *Plasmodium*, it is critical to test whether ML10 also blocks *Toxoplasma*, *Cryptosporidium*, and *Babesia* infectivity through inhibition of PKG.

One drawback of specific PKG inhibitors is their slow killing rate in parasite reduction ratio assays similar to that of pyrimethamine (110, 113, 114). In *Toxoplasma*, loss of PKG does not inhibit replication (72); therefore, it takes time for PKG inhibition to kill parasites by antagonizing egress and invasion. In *Plasmodium*, PKG is active within a very narrow temporal window of asexual blood stage development just prior to egress, which is thought to persist until the completion of invasion (36, 115, 116). Specific PKG inhibitors have no activity against parasites outside of this window in asexual blood stages (115). Similarly, PKG is only required during a 10-second time window upon exposure of gametocytes to xanthurenic acid and a rise in extracellular pH upon ingestion by a mosquito (31). Untimely activation of PKG through PDE inhibition could therefore lead to premature egress of all apicomplexans, ablating parasite proliferation and pathogenesis. More research is thus needed to better understand how interfering with cyclic nucleotide signaling could be exploited for therapeutic development.

### The challenge of specificity

Most molecules that are currently used against apicomplexan parasites have been developed to target human PDEs, which could lead to problems regarding specificity. Future work will be required to optimize pharmacological inhibitors of apicomplexan PDEs, which can be done by using human PDE scaffolds and testing different modifications regarding their antiparasitic activity. This “inverted silver bullet” approach (32) has been applied to the structural modification of tadalafil to find structural analogs with antiplasmodial activity, starting from the premise that differences in structure-activity relationship between human PDE5 inhibition and antiplasmodial activity can be exploited to improve the selectivity of drug candidates (117, 118), but further work and better structural knowledge of *Plasmodium* PDEs will be required for this. Our phylogenetic analysis indicates that all four *Plasmodium* PDEs are distantly related to their human counterpart suggesting that the development of selective inhibitors against *Plasmodium* PDEs is possible. Specificities of PDE inhibitors, however, can vary across different *Plasmodium* species. For example, the non-specific PDE inhibitor IBMX does not inhibit PDE activity in *P. falciparum* blood stages but increased cAMP levels in rodent parasites, precisely in *P. berghei* and *P. yoelii* sporozoites (46) and *Plasmodium chabaudi* trophozoites (119). Likewise, the development of highly specific PDE inhibitors will be useful in other parasites with reduced PDE repertoires, like *Babesia* and *Cryptosporidium*. In apicomplexans with expanded PDE repertoires (i.e., coccidians), inhibitors that target multiple PDEs at once will be more likely to kill these parasites. Additionally, coccidian PDEs’ inhibitors specific to bradyzoite development and maintenance will be useful in clearing the chronic infection of coccidian diseases. However, specific inhibition of critical tachyzoite PDEs, such as PDE2 in *Toxoplasma*, would likely kill these parasites as well (66).

### Emergence of resistance: the elephant in the room

A possible challenge for novel PDE-inhibiting medications is drug resistance that has emerged for almost all known antimalarials. It currently remains unknown how parasites may evolve against PDE inhibition, either via mutations affecting drug binding or penetrance or by modulating pathways upstream or downstream of cyclic nucleotide degradation. However, simultaneous inhibition of multiple apicomplexan PDEs would make drug resistance less likely to emerge from on-target mutation naturally.

## CONCLUDING REMARKS AND OUTLOOK

PDEs are undoubtedly required for parasitism of host cells by apicomplexans and make attractive targets for therapeutic development. Advancements in chemical- and reverse genetics in the “omics” era of biological research have greatly accelerated PDE research, enabling discoveries of essential PDE functions in a variety of apicomplexan species and life stages. However, the role of PDEs in most apicomplexan life stages is still limited to expression studies at best. Even the most well-studied apicomplexan PDEs lack functional information at precise cycle transitions due to a lack of effective live-cell cyclic nucleotide reporters. Development of genetically encoded indicators of cGMP and cAMP, specifically for apicomplexan parasites, will enable spatiotemporal investigations of PDE function in real time. Furthermore, structural information for apicomplexan PDEs is limited to motif searches and modeling predictions. Solving the structures of apicomplexan PDEs will reveal mechanisms of cNMP binding and hydrolysis and inform rational drug design for PDE inhibition. Finally, understanding how PDE activity is modulated by kinase phosphorylation may reveal feed-forward or feedback loops in cNMP signaling or cross talk between cNMP and other signaling pathways. These avenues of exploration represent the next frontier in apicomplexan PDE research.

## SUPPLEMENTAL MATERIAL

Supplemental File 1 mbio.03056-23-s0001.pdf Full PDE phylogenetic tree. Related to Figure 1.

ASM does not own the copyrights to Supplemental Material that may be linked to, or accessed through, an article. The authors have granted ASM a non-exclusive, world-wide license to publish the Supplemental Material files. Please contact the corresponding author directly for reuse.
