# Erratum for Moss et al., "Apicomplexan phosphodiesterases in cyclic nucleotide turnover: conservation, function, and therapeutic potential"

**DOI:** 10.1128/mbio.00002-26

**Published:** 2026-01-21

**Authors:** William J. Moss, Lorenzo Brusini, Ronja Kuehnel, Mathieu Brochet, Kevin M. Brown

## ERRATUM

Volume 15, no. 2, e03056-23, 2024, https://doi.org/10.1128/mbio.03056-23. Page 19, Acknowledgments, paragraph 2: “5P20GM143973” should read “5P20GM134973.”

