# Supplementary material for: Apicomplexan phosphodiesterases in cyclic nucleotide turnover: conservation, function, and therapeutic potential
Source: mBio. 2023 Dec 22;15(2):e03056-23. doi: 10.1128/mbio.03056-23 (PMC10865986; doi:10.1128/mbio.03056-23)

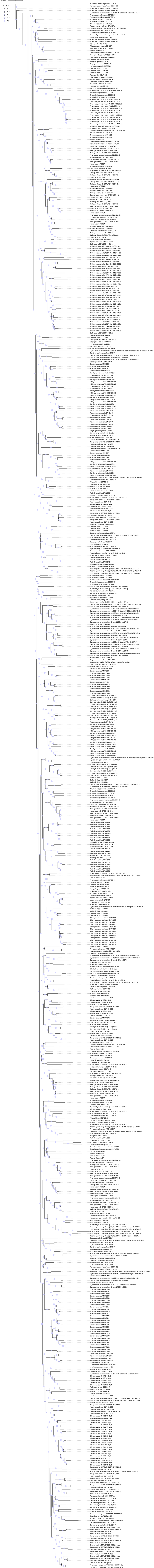

Supplement: Supplemental File 1 — Full PDE phylogenetic tree. Related to Figure 1. [file mbio.03056-23-s0001.pdf]
